# Earth Science, Art and Coastal Engineering at the Seaside: Envisioning an Open Exploratorium or Geo-Promenade at Weston-super-Mare, Somerset, United Kingdom

**DOI:** 10.1007/s12371-022-00745-1

**Published:** 2022-10-20

**Authors:** W. Brian Whalley

**Affiliations:** Department of Geography, University of Sheffield, Sheffield, UK

**Keywords:** Exploratorium, Exploratory learning, Geology, Geomorphology, Geotourism, Geo-promenade, Public art, Cognition, Decision making, Behavioural environment

## Abstract

The paper outlines how an outdoor ‘exploratorium’ can be curated to place field observations, together with some knowledge of the local environment, in a manner that relates to geology, geomorphology and artistic aesthetics. The exploratorium can also be used to help explain what is seen, touched or felt as part of ‘sensory geology’. The locations used are on an accessible walk, a geo-promenade, along the sea front of a coastal town, Weston-super-Mare, England. Sites, such as a sea wall and recent engineering structures, are linked to flood prevention measures and sculpture and stone promenade furniture relate to various aspects of geoheritage. Notions of consilience and decision-making in the behavioural environment are introduced, together with ways in which perceptions of the landscape can be shared with visually impaired visitors. Notes are provided to illustrate the additional information (metadata) that might be supplied in constructing a geo-promenade. The use of mobile technologies for recording observations, providing locations (using decimal Latitude Longitude designations) and general information is also discussed with respect to accessible visits. Attention is also given to cognition and educational models of experiential discussion and knowledge sharing that can be used in exploratoria and geoheritage in general.

## Introduction and Background

Dowling and Newsome (2018, 1) define geotourism as ‘tourism based on geological features’. Here I explore aspects of a tourist’s viewpoint of a coastal seascape that includes not only a variety of geological and geomorphological features (geosites) but also art, sculptures, monumental features and other human artefacts such as engineering features. The paper is based on constructing a geoheritage-based ‘exploratorium’, a ‘geo-promenade’, where participants make observations and ask questions about the landscape and its components. Visitors may see features and phenomena from several points of view: geological-environmental, artistic, economic, social and behavioural. The paper places these aspects of a geo-promenade into a general pedagogic framework of exploratory learning that has been termed ‘rhizomatic education’ (Cormier 2008), where learning is most effective when participants discuss and react to evolving circumstances and may re-define tasks and questions. Such questions can also arise in planning decisions, for example when considering energy supplies and climate change, and where geo-environmental concerns may relate to economics as well as aesthetics. The geo-promenade envisaged here brings some of these concerns to visitors’ attention by exploring a range of issues connected with earth sciences.

**Fig. 1:**
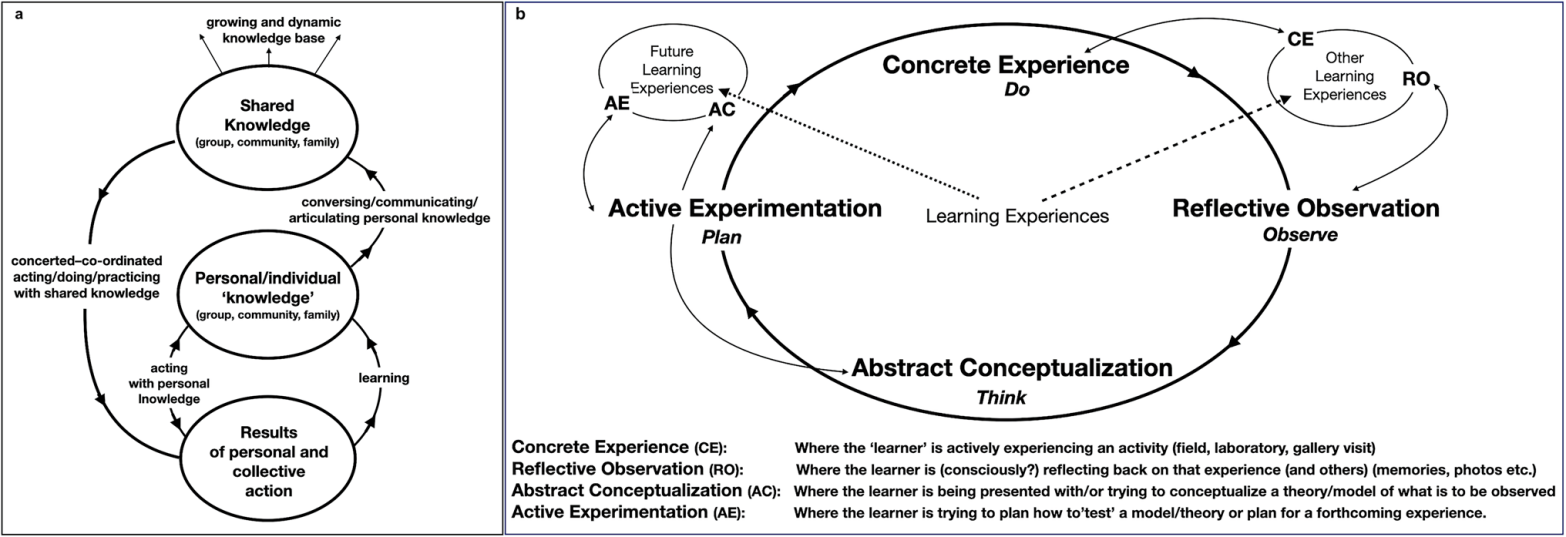
**a** Object-people, situative learning, relationships according to De Freitas and Neumann (2009) concerned with sharing knowledge and building and sharing communities of practice. **b** Healey and Jenkins (2000) experiential learning cycle, concerned with cognition and experiential learning, modified by adding other learning experiences

Loosely defined, exploratory learning is an approach to teaching and training that encourages the learner to explore and experiment to uncover relationships between objects and people (De Freitas and Neumann 2009 and Fig. 1a). It is based on constructivist theories of learning and teaching stemming from the Kolb’s experiential learning cycle of Healey and Jenkins (2000 and Fig. 1b) with little or no focus on didactic or formal learning but includes experience, exploration, reflection, forming abstract concepts and testing in different situations. Exploratory learning provides the basis for public exhibitions such as the *We The Curious* (formerly *@Bristol* and the *Exploratorium*) at Bristol, UK, or the ‘Exploratorium’ at Piers 15 and 17, and the California Academy of Sciences in Golden Gate Park, both in San Francisco, USA, and the *Questacon* in Canberra, Australia. In the UK, examples of subject related exploratoria include *Catalyst* in Widnes (chemistry) and the *Camera Obscura* in Edinburgh (optical illusions).

These metacognitive approaches help to set out material for a ‘geo-promenade’ and establish links with learners (or visitors/participants) that are not rigid structures but suggest various ways of bringing a diversity of experiences to the sites. The experiences described in the present paper can be used to provide indications of what visitors might see and to provide supporting material for ‘learners’ of whatever age group and experience who are unlikely to know much geoscience. The geosites show how important earth sciences are to the visual-aesthetic as well as behavioural environments.

Although primarily seen as ‘for kids’, exploratoria provide learning opportunities for *any* person and should thus be inclusive, as family visits for example. Exploratoria are often science based, despite sometimes having an ‘art’ content or theme. This raises questions, especially for artists using science as inspiration or exploration, as to the meaning of the terms ‘art’ and ‘science’ or indeed phenomenological approaches to landscapes (Hawkins 2018; Whalley 2022). This paper is not directly concerned with such distinctions, but rather explores linkages between active, or exploratory, learning and breaks down the formalities of subject areas and disciplines in the manner of E.O. Wilson’s ‘consilience’ (1998). For example, the vision of the *Outback Exploratorium* (Emerald Queensland, Australia) is ‘to inspire and ignite young minds in the wonders of S.T.E.A.M. (Science, Technology, Engineering, Arts, Mathematics).’ This is, roughly, in keeping with Prensky (2017) ‘new paradigm of the curriculum’ by suggesting that an educationally informal approach to geoheritage and geoscience is important. Incorporating aspects of public art and also by viewing elements of landscape as art brings in open engagement (Reyes 2016) to the ‘urban–nature’ interface. Interfaces abound in geo- and cultural-heritage studies (Pijet-Migoń and Migoń 2022), this geo-promenade allows some of these to be explored, particularly those related to ‘art and craft’ and aesthetics. However, there are substantial differences in what is meant even by ‘landscape’ and the distinction between ecological and semiotic discourses (Lindström et al. 2018). It is the former that is largely followed in this paper.

Entrance fees for the formal exploratoria mentioned above are not cheap and rarely gratis. This paper shows how an exploratorium might be set up, using existing facilities in a city, town or village for little or no expense. From this point of view, it is ‘open’. It does not *require* modern technology although ‘smart’ devices might be useful beyond ‘social interaction’ as shown later. In particular, I suggest how imagination, creativity and a little knowledge can be used to make self-guided explorations for individuals, a family on holiday or school groups. Sensory interaction and observation are explicit as a means of producing inclusivity in learning beyond the confines of academic subjects. In this sense too, it is ‘open’, not formal education, outdoors and provides educational opportunities for people with diverse handicaps. Learning at home has become part of the Covid-19 lock down; open exploratoria, as suggested here, are an extension of this, and could perhaps be promoted by a local museum and specialist societies in conjunction with local tourist offices. The paper investigates what the town of Weston-super-Mare has to offer as an example. It sets out ‘stops’, geosites, at identified locations where things to see, do or explore can be found. This is as any visitor to a museum or art gallery might experience — except that it is informal, in the open air and is accessible to individuals or groups with push or wheelchair. The notes in this paper might be used as extra information (metadata) that could be added to this geo-promenade.

Despite mass formal education, knowledge is cognitive and personal (Fig. 1a, b);

Your knowledge alters how you see things. The things you see become part of your knowledge. Your knowledge alters how you see things. You can never see the things themselves. (Stone 2012, 155)

This paper presents examples of interactions of some selected observable features, mostly in plain view. In particular, it examines appreciation of aspects of geology, geomorphology and sculpture with some basic aesthetic processes in a coastal setting. The paper also links aspects of perception and cognition of environmental artefacts, natural and man-made, within a domain of explanation, problem solving and interdisciplinarity. This can be achieved across science and the visual and haptic arts.

In many educational systems, learning is curriculum or subject-based, especially for students in secondary/high schools and higher education. However, educational journeys may benefit greatly when learning is out of the formal classroom context, perhaps on holiday. In particular, inquisitiveness can be stimulated by exploring linkages between objects, people, places and events (Fig. 1). The attributes of observation and the ability to cross disciplinary boundaries are difficult to teach formally, but ‘out-of-class’ activities can promote knowledge discovery at diverse levels. This paper shows how this might be accomplished by observing ‘the geo’ in local and historical landscapes by linking to artworks and decision-making in a time of climate and environmental changes.

Of recent years, the STEM subjects (Science, Technology, Engineering, Mathematics) have been used as a suggestion for economic progress, while in the UK, SHAPE (Social sciences, Humanities and the Arts for People and the Economy) has been proposed and in the USA, STEAM (Science, Technology, Engineering, Arts, Mathematics) has been employed (especially for k12 education). However, I prefer to mix and integrate ‘knowledge’, better to tackle the Fourth Industrial Revolution (4IR), that is via STEAMS (Science, Technology, Economics, Arts, Mathematics, Social sciences) with respect to consilience. This idea is extended in a post-Covid-19 higher education setting by Whalley et al. (2021). As well as the formulation of an exploratorium, the cognitive aspects mentioned also relate to knowledge integration in geoscience education (Kastens and Manduca 2012) that can be related to artistic endeavours associated with geoheritage. Geoscience is currently involved in many planning decisions; for example, hydrocarbon extraction, renewable energy generation, coastal erosion and flood prevention measures, where improved public knowledge of geoscience should enhance understanding of the issues involved. Notes provide extra, contextual, information to add to knowledge bases (Fig. 1).

## Aim and Objectives

This paper investigates how an outdoor exploratorium, a geo-promenade, can be constructed to link and integrate observations, both visual and tactile, to some geological, geomorphological engineering and artistic entities that are part of a location’s character. The paper also shows how aspects of science/society/sustainability as part of geoheritage (Sztein and Whitacre 2021) can be brought together at a populous locality with reference to education, social awareness and aesthetics. After a brief overview of the nature of ‘the exotic’ and the individuality of knowledge, the paper indicates how:

i. An exploratorium can provide access to informal points of observation, information, discussion, education and participation.
ii. Observations can convey information about temporal events and change over annual, decadal, centennial and millennial time spans.
iii. Observations for the inclusion of visually impaired, mobility-restricted and neurodivergent may be introduced into such experiences. ‘Observation’ here also relates to touch, hearing, taste and smell.
iv. An exploratorium can be made to be a flexible, outdoor experience (or field trip) that can be used by participants to make their own storylines about a visit or holiday, perhaps recorded on smart devices, to produce inclusive and shared experiences.
v. An exploratorium can be used at a variety of age levels from children onwards and might include research on topics of educational interest for students and teachers. It should be capable of being followed by an individual or small group.

An accompanying guide to the locations visited on the geo-promenade could be produced on paper, although dissemination by internet-based links to mobile devices allows for on-demand information. As an exploration, a guide can be started and stopped in any locality and followed by people with wheelchairs and pushchairs and in (more or less) any weather conditions. Locations may be viewed by individuals or small groups as a self-guided tour. Cognition is enhanced in a variety of ways, across subjects and interests, by taking people out of their comfort zones. The geo-promenade is real life but aspects of virtual, or augmented, reality might be brought into play to extend experiences (Fig. 1).

## Observation, Explanation and Judgements

For most of us, visual observations are a major way in which we communicate with the world. A six-month-old child is looking around and making observations and neural connections well before they can speak. Yet they observe and take note and can communicate by signing. When they are able to speak, they may not have the knowledge or vocabulary to ask more than, ‘what’s that?’; later perhaps, ‘what’s it for?’ or ‘how does it work?’ or perhaps ‘what does it mean?’. We give ‘explanations’ which may suffice at that time, at home, in the street or in school. These are what Stewart and Cohen (1997) have called, ‘lies to children’, the explanation will do, or will have to do, because we do not yet know enough (Kastens and Chayes 2011). In fact, such questions occur all the way through life and should be accommodated in any educational setting, formal (as in school or higher education) or informal (such as watching a TV documentary or during a museum visit). The questions are not limited to arts or sciences but should be inclusive (Gamwell 2020, Gamwell 2016; Wilson 2010; Leeder and Lawlor 2017). Queries also apply to political debate and decision making from local to national agendas. In the UK at present, debates include wind turbine farms and tidal barrage schemes for electricity generation and transmission and hydrocarbon extraction, their aesthetics and impacts on rural economies.

We need guides to link observations to existing knowledge bases through interpretation or metadata to show inter-connections. Many TV documentaries, although providing vicarious observations in remote or inaccessible locations, often fail to satisfy the need for ‘explanation’ let alone ‘understanding’. They are for entertainment rather than education. On the spot interrogation of Wikipedia by a mobile device may help to plug the knowledge gap. However, such factual (declarative) knowledge needs to be brought together with tacit (or personal) knowledge as well as skills and abilities to develop our personal knowledge management systems. Such personalization is what educational metacognition aims to accomplish (Reigeluth et al. 2017). This is also relevant to geoscience education with respect to learning progression: ‘It is important to differentiate between scientifically inaccurate ideas that are conceptually unproductive and understandings that are inaccurate, yet productive, and that can foster learning of more sophisticated understandings’ (Duncan and Rivet 2013 396).

Out of classroom activities for students of whatever age, from fieldwork to art gallery or museum visits, provide immediacy and opportunities for explanation via exploration. Increasingly, and across many disciplines, observations, information and decision making are seen through a pinhole, if not lens, of the anthropocene. Issues relating to geographical studies and the anthropocene have been discussed by Castree (2014, 466); ‘Given that Geography is among the few subjects disposed to studying humanity’s relationships with its ‘natural’ and created environments, it may have something to say about *both* the ‘physical’ and the ‘human’ dimensions of life after the Holocene’. But further, social science and psychology show that the manner that people perceive and react to environmental and social change is both varied and contingent. They can, ‘elucidate the value judgements in most things that people do, including by experts across all fields’ (Castree 2017, 289). Such expert areas include art (Davis and Turpin 2015) and ecocriticism (Clark 2015) as well as earth sciences (Tooth et al. 2016). On the negative side, geology has been perceived in the UK as detrimental to the environment (such as through exploitation of mineral and hydrocarbon resources). Discussion of global heating research and sustainable geoscience needs to be promoted (Stewart and Gill 2017; Jackson 2020) and with reference to geoheritage (Gray 2019).

Observing and understanding are necessary attributes before explaining something to non-specialists, as well as crossing disciplines in sciences, arts and engineering. In particular, observing, involving rather more than looking and describing, might also be invoked by the police, lawyers, novelists and journalists. In formal education, these cognitive terms or functions might be implemented on a ‘novice-to-expert’ trajectory. What is less easy to instil is creativity and appreciation of a range of cognitive processes whereby all humans perceive, interpret and even alter the world (Csikszentmihalyi 1996).

Stewart (2008) discusses environmental education pedagogy by way of ‘reading the landscape’ with reference to Australia, suggesting that,

There is no single history of a place: different stories reveal different values, attitudes, behaviour and impacts. Without historical stories place(s) become meaningless, featureless backdrops upon which new cultural and environmental injustices may be written (Stewart 2008, 94).

For example, Zhang et al. (2022) have explored the ‘natural beauty’ and aeshetics of some World Heritage karst sites in areas which to many people are ‘exotic’, that is unfamiliar. Yet, exotic may be anywhere and explored in the outdoors; participation can be enjoyed by anyone and not vicariously via a TV producer. Engaging with the exotic can also be associated with presenting ideas and scenes from alternative points of view and can elicit creativity. This diversity can relate to the arts, associated with an ‘exploratory’ approach linked to climate and environmental change and also to decision-making in the anthropocene. But in observing, we need to be inclusive and include mobility, those who are visually impaired as well as with varying degrees of sensory deprivation and neuro-divergence. A further issue relates to ethical issues, such as ‘carbon footprint’. Can we bring the ‘exotic’ close to home (in this case for the UK) without excessive travel? Visiting the exotic is relative. Thomas Moran’s paintings of ‘The Grand Canyon of the Yellowstone’ (1872) and ‘The Chasm of the Colorado’ (1873–4) brought the exotic geology and landscapes of Ferdinand Hayden’s ‘Western Surveys’ to the east coast of the USA. Where, of the Yellowstone, Moran, ‘marshaled every aspect of this picture – the subject, the forms, the technique – to convey the transforming powers of time and the forces of nature. His concern with the geological history of the site is evident’ (Bedell 2001, 132).

‘‘When something is new to us, we treat it as an experience. We feel that our senses are awake and clear. We are alive’’. Jasper Johns, quoted on a plaque near the entrance to the *Exploratorium*, San Francisco.

## Weston-super-Mare as an ‘Exotic’ Holiday Location

**Fig. 2:**
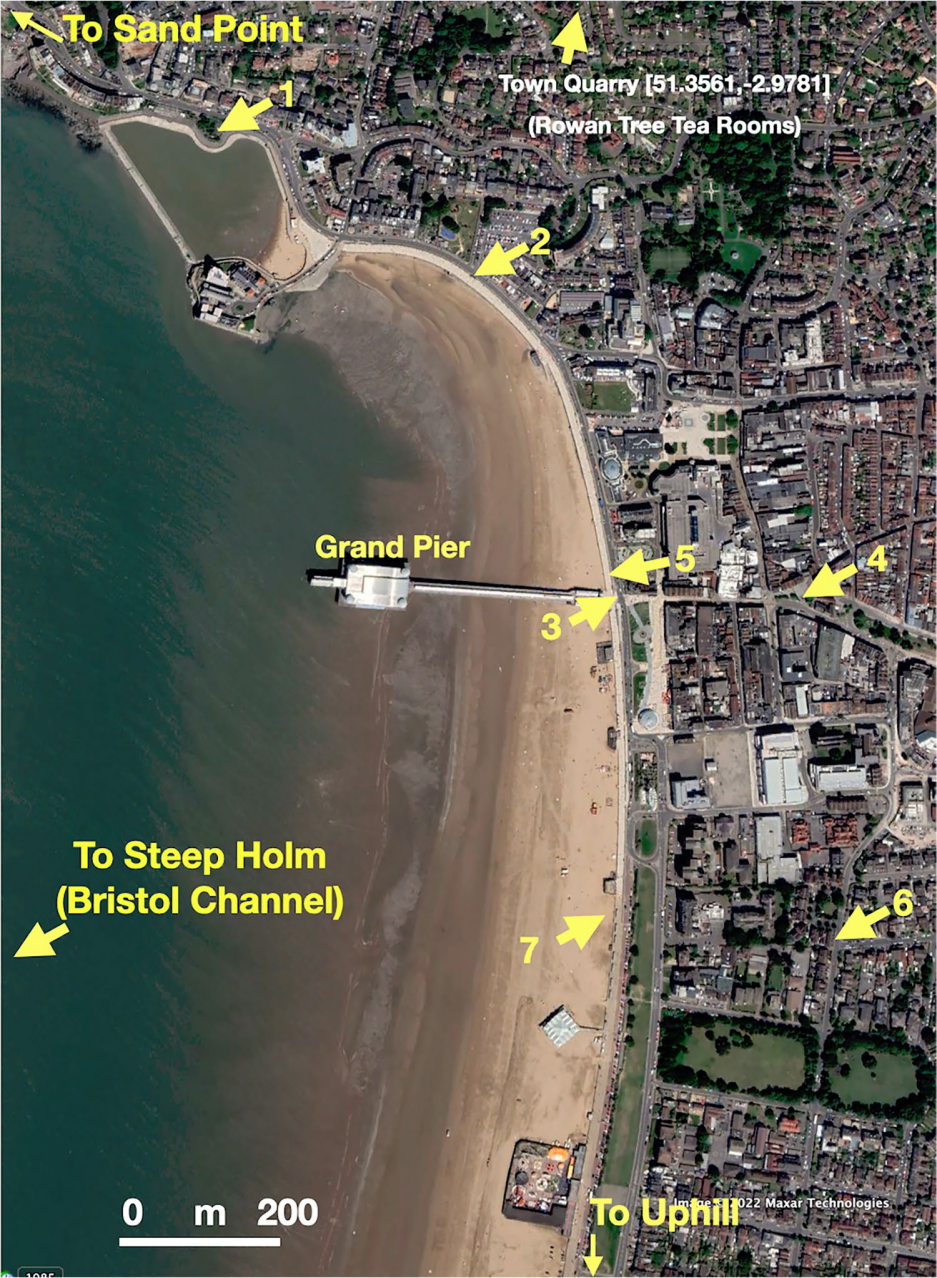
Google Earth (GE) view of the north part of Weston-super-Mare. Numbered arrows refer to locations in the text with decimal Latitude Longitude (dLL) locations: 1. Holm [51.353528,-2.989751] (Fig. 7). 2. John Maine’s *Weston Arch* [51.35116,-2.9845]. 3. Splash wall gates [51.34763,-2.98158] (Fig. 4). 4. Wolfgang Buttress’ *Silica* [51.34764,-2.97807]. 5. Grand pier and weathered wall [51.34762,-2.98166] (Fig. 5b). 6. Eddington plaque [51.34363,-2.97734]. 7. Sand sculptures [51.3437,-2.9817] 2016 image. Image ©Google Earth

Weston-super-Mare in Somerset, south-west UK, is a typical Victorian holiday town growing as a result of railways with a pier, beach and ‘attractions’ (Brodie et al. 2019; Butt 2018). Railways in the 19^th^ century brought many tourists to the seaside for the first time. WGS84 co-ordinates [51.34762,-2.98166] centre on the Grand Pier entrance, and Google Earth, Street view and OpenStreetMap can be used to visit locations mentioned in this paper (Fig. 2). (The dLL, [decimal Latitude Longitude] format can be pasted directly into these applications.) If the visitor, or indeed resident, looks around there is much to see of historic and artistic interest^1^. An example of a multi-purpose sculpture in the town is ‘Silica’^2^ (by Wolfgang Buttress, built 2007). The *Wonders of Weston* are part of the EU Sea Change project^3^ but other ‘observation stops’ mentioned below are geological-geomorphological and may also require an observer’s eye. That is, demanding of attention, not just being told ‘that’s art’ or ‘that’s geology’. These are items (geo-)visitors can explore as ‘found’ objects worthy of interest, observation, study and explanation (Fig. 1).

We can progress from observing features to making creative interpretations involving sight and touch. Smartphones or tablets are not essential for this fieldtrip, although they might be useful for putting together a ‘learning experience’ by linking the ‘geo’ with ‘humanities’ as linked snapshots or video or by creating sketches on notes. Observation is encouraged by standing still and sketching rather than taking a photograph and moving on (Whalley 2019; Forget 2021). Simple visual aids, hand lens and binoculars, assist inspection, although they may be incorporated on smartphones as investigatory tools and where notes can be pasted into say, a geological scrapbook of a visit.

The late Stephen Hawking suggested ‘Remember to look up at the stars and not down at your feet’ (McGowan 2018). As well as having feet firmly on the ground, observations generally go from where we are at any time and from where we have been — our previous experiences. This invokes aspects of time as well as memory. All of these will be different for each of us; no matter how old or young, of whatever educational background. Bodily impediments can be turned to advantage to provide different ways of looking (Ward 2014) but they need to be accommodated in the questions asked and objects presented as well as with respect to mobility. To Hawking can be added, ‘You will understand the true spirit neither of science nor of religion unless seeking is placed in the forefront’ (Eddington 1929). Sir Arthur Stanley Eddington, astrophysicist, famous for work on relativity at the time of Einstein and one-time resident of Weston^4^. Astronomers and cosmologists, as well as geoscientists, deal with observations over space and time that may present difficulties for casual visitors to landscapes.

The promenade suggested here does not have to be done sequentially or in any direction or at any time or state of tide. Indeed, the visitor does not have to be present, just observe. It shows how observation and enquiry cross disciplines educationally. Although Weston, with all its individual characteristics of time and place, is the subject of this geo-promenade, any location could be used for this multidisciplinary approach.

## Public Art: Sand Sculpture Exhibitions at Weston-super-Mare

**Fig. 3:**
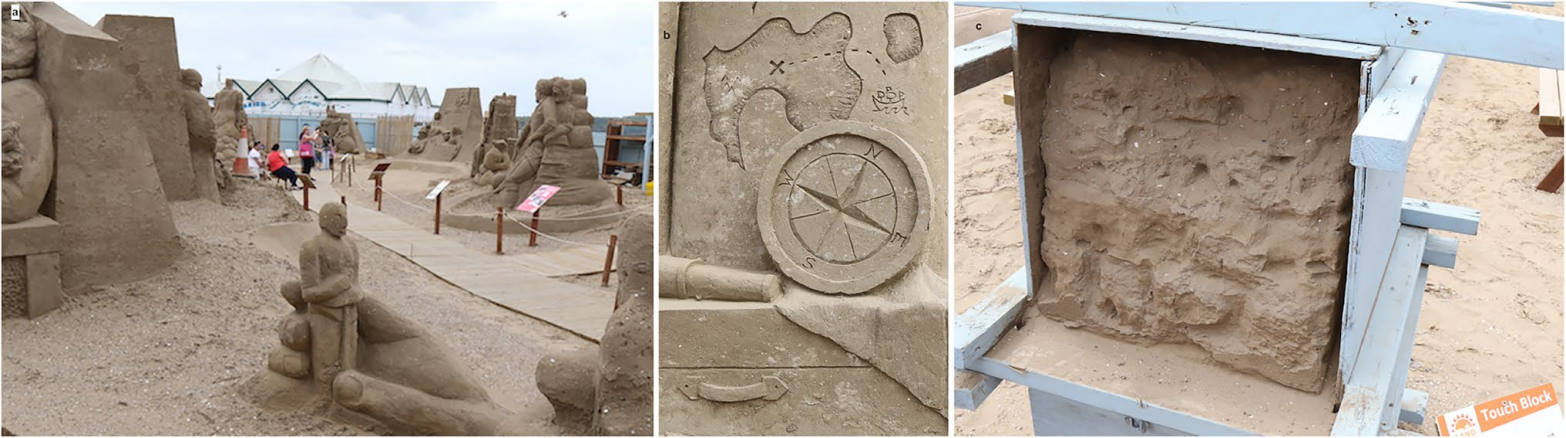
**a** Part of the sand sculpture exhibition 2016 showing the scale of some of the sculptures. **b** A detail of one sand carving. **c** Demonstration ‘touch block’ of the compacted sand. Photos: ©W.B. Whalley, 2022 CC BY-SA 4.0

The first example links observations of sculpted sand, its texture and mechanical behaviour. Public art is not necessarily permanent. In 2015 the artist ‘Banksy’ (originally from Bristol, 20 km north) opened the temporary, and once only, installation or ‘bemusement park’, ‘Dismaland’ at Weston^5^. In the summer of 2022, an installation, ‘See Monster’ — a decommissioned North Sea exploration platform — will visit Weston as a piece of explorable public art. Annually from 2005, although not post-Covid-19, Weston has hosted a themed sand sculpture festival on the beach^6^. The images here are of artists’ sculptures made from the beach sand (Fig. 3), which engender questions about the nature of this ubiquitous material.

The faces, detail and sharp edges do not blur over time despite no adhesives being used. The reasons beach sand can behave like this can be explained by simple sedimentology. A high bulk density of the beach sand is achieved by tamping into blocks (Fig. 3c) to minimise void space. A little moisture is retained to promote ‘apparent cohesion’ and increase shear strength. Fracture of sand grain edges during tamping may help to fill voids and increase bulk density. The longevity of the sand sculptures at high slope angles contrasts with the approximately 30° slopes seen on dry sand dune ‘slip faces’. Sand dunes occur in many coastal sites, at Weston, dunes occur to the south of the bay, but the esplanade was originally built, about 1826, on top of sand dunes. The original esplanade can be seen in a painting from about 1860, reproduced in Brodie et al. (2019).

Additional earth science concepts and questions that might be introduced and discussed as part of the learning experience at this locality include what are ‘shear strength’ and ‘cohesion’, has mud a shear strength, do sand grains differ on beaches and in sand dunes, do sand dunes move?

## Sea Defences: Geoheritage and Engineering

**Fig. 4:**
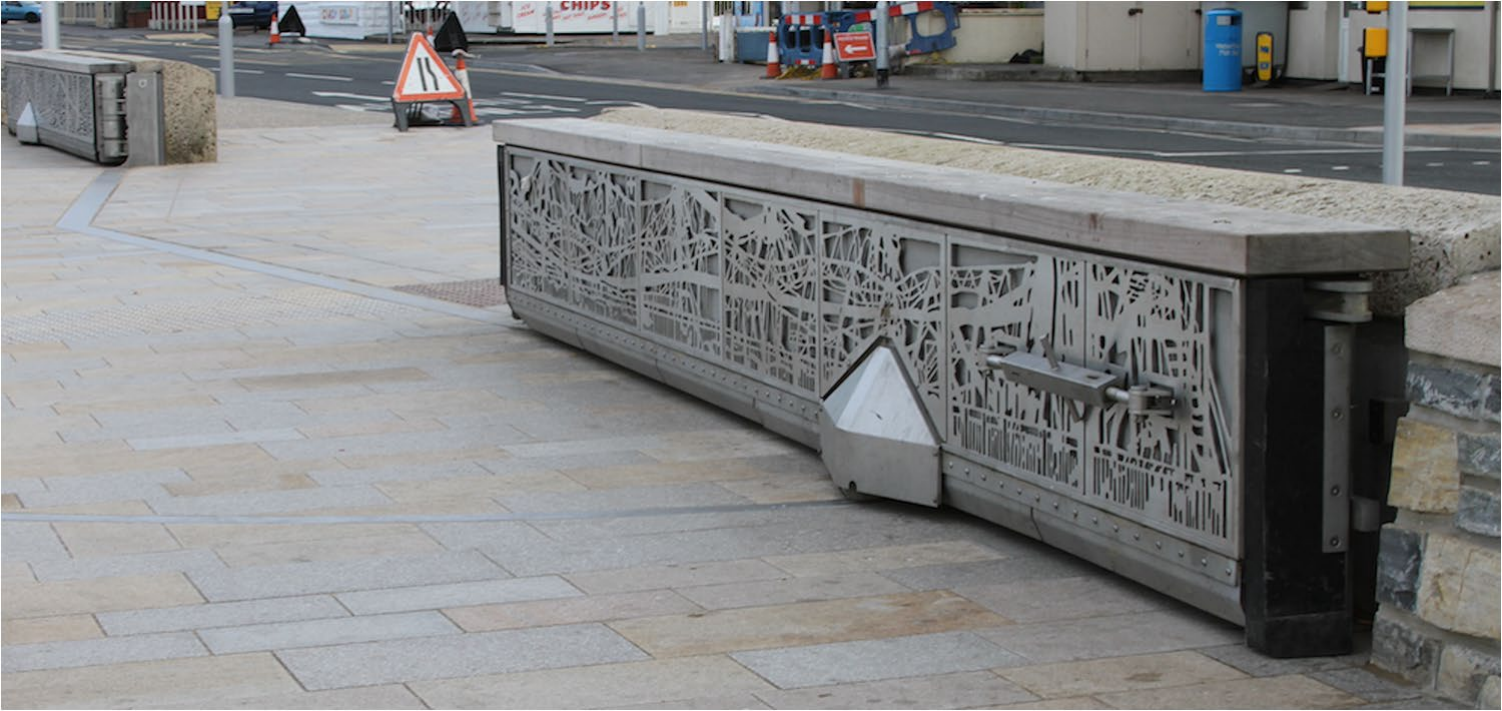
One of the twelve flood gates on the esplanade’s second, storm surge, ‘splash wall’ with part of a shopping area opening behind. This is the double gate outside the Grand Pier, the gate’s pivot is in the centre (Fig. 2). Photo: ©W.B. Whalley, 2022 CC BY-SA 4.0

This example shows how some effects of climate change can be mitigated by discretely-placed engineering structures. The tidal range in the Bristol Channel may be as much as 14 m and, being west-facing and with the low-lying heart of the town exposed to the coast, there has been occasional flooding in Weston-super-Mare. In December 1981 there was gale damage to the promenade and the sea defences at Sand Bay were overtopped by the storm tidal surge. A sea ‘defence’ scheme has now been incorporated into the promenade area^7^. The defences are not obvious because a secondary sea wall, or ‘splash’ wall, between the sea-front road (Royal Parade/Knightstone Road) and seafront promenade is pierced in several places by walk-through areas. When necessary, the gates, normally folded into the wall, are jacked into place. Several of the gates themselves are attractively decorated (Fig. 4). The splash wall used reinforced concrete, clad on both sides with a combination of Blue Lias and reclaimed local stone, (probably Lower Carboniferous, Clifton Down Limestone).

To the south of the main civic area, the esplanade road (Marine Drive) now goes over a substantial ramp that effectively continues the sea wall. These defences were funded as part of the *Sea Change* project to protect the town centre. Google Earth images for 2009 show this work under construction.

Some questions for discussion at this location might include how do tides ‘work, what are storm surges, how often do these occur and might the town still be flooded, what happens in times of rising sea levels, how much is sea-level rising here, is the storm tidal surge of December 1981 likely to be repeated?

## Sea Wall and Stone Weathering

**Fig. 5:**
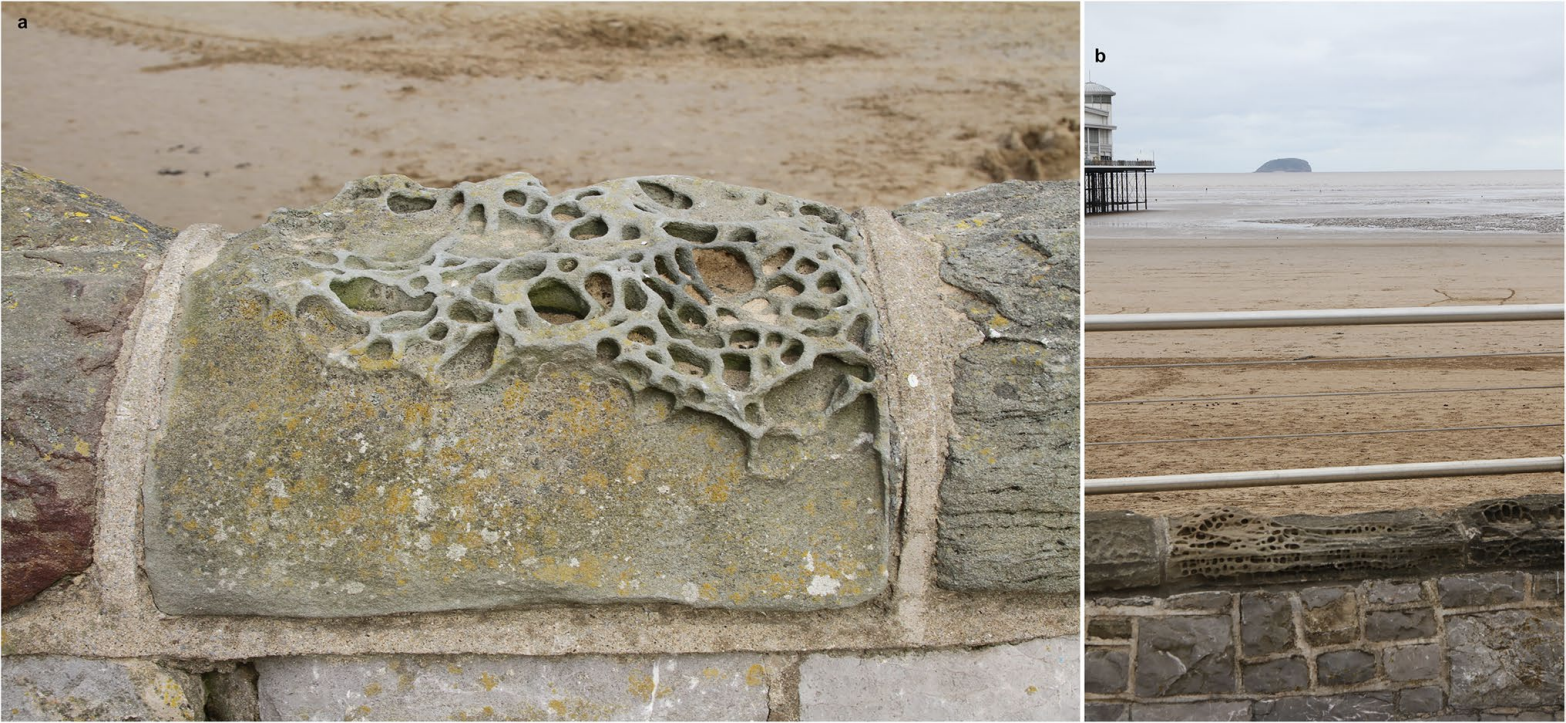
**a** Coping stone of Forest of Dean sandstone differentially eroded by salt weathering to produce ‘honeycomb’ or ‘alveolar’ weathering’. Differences in the coping stone’s response to weathering can be seen left and right. **b** Honeycomb-weathered sandstone coping on the sea wall with recently (2016) added stainless steel rails. A portion of the Grand Pier can be seen on the left with Steep Holm on the horizon. The rails contrast in colour as well as texture with the stone wall, which is the more aesthetically pleasing? Photos: ©W.B Whalley, 2022 CC BY-SA 4.0

Observing contrasts of features, how they look and feel, promotes discussion of such material differences. Visitors may not have noticed the gates on the second sea defence wall. Perhaps more obvious is the occasional honeycomb (or alveolar) weathering on the top of the sea wall above the beach around the esplanade (Fig. 5). The sea wall was completed in 1888 with Forest of Dean sandstones (Devonian) being used for the coping stones.

These weathering features were discussed by Mottershead (1994) who examined their spatial variation in areas around the north side of the bay. At first sight they appear to be architectural ‘vermicular rustication’ on the surface, but the features are natural not man-made, although developed from the original patterning of the stone capping. Mottershead attributed the features, as have others, to salt weathering; spray enters the sandstone pores, water evaporates and salt crystals grow and expand. This weakens the sandstone mass and releases grains then washed or blown away. The spatial variation of the weathering intensity, as shown by the development of the honeycombs, was mapped by Mottershead and related to distance from the sea, height above the sea and aspect as well protection from buildings on the wall. Bruthans et al. (2018) have recently discussed the formation of such weathering features^8^. Since Mottershead made his observations, the coping stones have been removed and replaced, in their original sequence as far as can be ascertained, so long-term comparison is still possible.

Stainless steel rails have now been bolted to the wall (Fig. 5b). This addition, presumably for ‘safety’ reasons, might seem to detract from the natural weathering of the stone. The stone weathering process, over some 130 years, increases roughness and contrasts, by eye and touch, with the smooth and shiny stainless steel as well as rusting iron railings seen elsewhere. Weathering and manufacturing artefacts contrast mixed media in material science and art and design. As well as visible texture, the difference between the two forms of fencing is tactile and can be touched by people in wheelchairs or push-chairs.

Some more geological questions for discussion might include do all rocks weather in that honeycomb manner? Why are some coping stones devoid of honeycombs, what is rust, how does stainless steel differ from iron?

## Steep Holm

Visual images are a major part of holidays and sightseeing as well as well as many aspects of earth science knowledge, ‘To read the landscape like a book, as well as to enjoy it as a picture’, (Crowe and Mitchell 1988, 3). The photographs in Crowe and Mitchell’s book have captions from an aesthetic viewpoint but rarely about locations or geology and geomorphology. Conversely, the book ‘Landscape, natural beauty and the arts’ (Kemal and Gaskell 1993) includes not one image! Nor does Wylie’s (2007) ‘Landscape’ show any of the artworks or scenery referred to. It includes an idea from Daniels (1989) that ‘landscapes are duplicitous’, ‘they tend to present themselves as ‘scenery’ or ‘nature’ in a way that obscures and masks the social and economic conditions that go into their making’ Wylie (2007, 104–5). The geo-promenade can help bring images, information about materials, metadata and cognitive experiences together with discussions about aesthetics (Zhang et al. 2022). Additionally, economics and decision making may also play an important part in planning considerations and decision making.

**Fig. 6:**
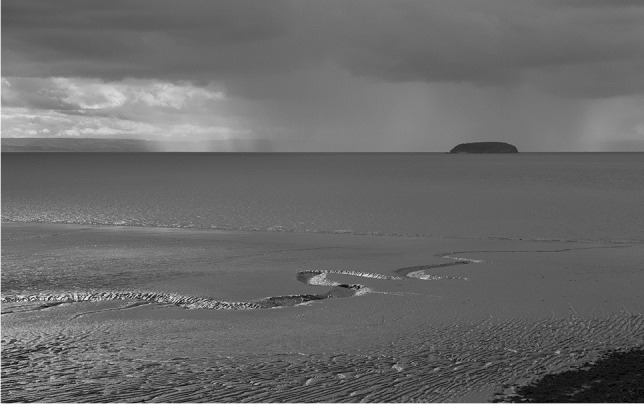
Steep Holm from Sand Point, North Somerset, September 2015. ‘Fourier-scape 1, squall approaching; View south-west from [51.388277,-2.978030] 17/09/15, 13.03 UTC’. Photo: ©W.B. Whalley, 2022 CC BY-SA ND 4.0

With changing lighting and sea conditions, the seaside always has interesting visual changes and contrasts. Figure 6 shows Steep Holm^9^, an island some 10 km off-shore [51.3398,-3.1084], usually visible from Weston and Sand Bay to the north. The island is composed of Lower Carboniferous limestone (350 Ma) and might be a continuation of the Mendip limestone at Brean Down [51.3257,-3.0228], although structures on the island suggest considerable folding perhaps, Variscan/Hercynian Orogeny (end Carboniferous). In close up, the white limestone, as well as the structures and bedding, is distinctive, although the cliffs are much covered in vegetation.

From the shore, Steep Holm tends to look dark, perhaps forbidding. Figure 6 was taken from Sand Point [51.388277,-2.978030] looking south-west over Sand Bay at low tide and with a squall over the sea. A monochrome image gives the landscape ‘atmosphere’ and contrasts the Sand Bay muds and sands at low water with the solid limestone of Steep Holm. Photography, as well as being creative, provides a focus on geological and geomorphological features and can also link to the history associated with Steep Holm. With a smartphone available, photography and imaginative use of post-processing can become an important tool for capturing observations as well as a ‘view’ and ‘atmosphere’. Visitors can link geological aspects of the scenery creatively. The title given is impersonal and only locates the scene, unique in time, atmospheric and geological conditions. The linking of visual imagery to landscape, whether as a geomorphological entity or artistic endeavour, provides a good opportunity to contest the duplicitous nature of landscapes.

Earth science queries abound here: where has all the rock between Weston and Steep Holm gone, how long did it take for this erosion to take place, where has mud in the bay come from, does it always stay there, are cliffs retreating in this area?

## Holm

**Fig. 7:**
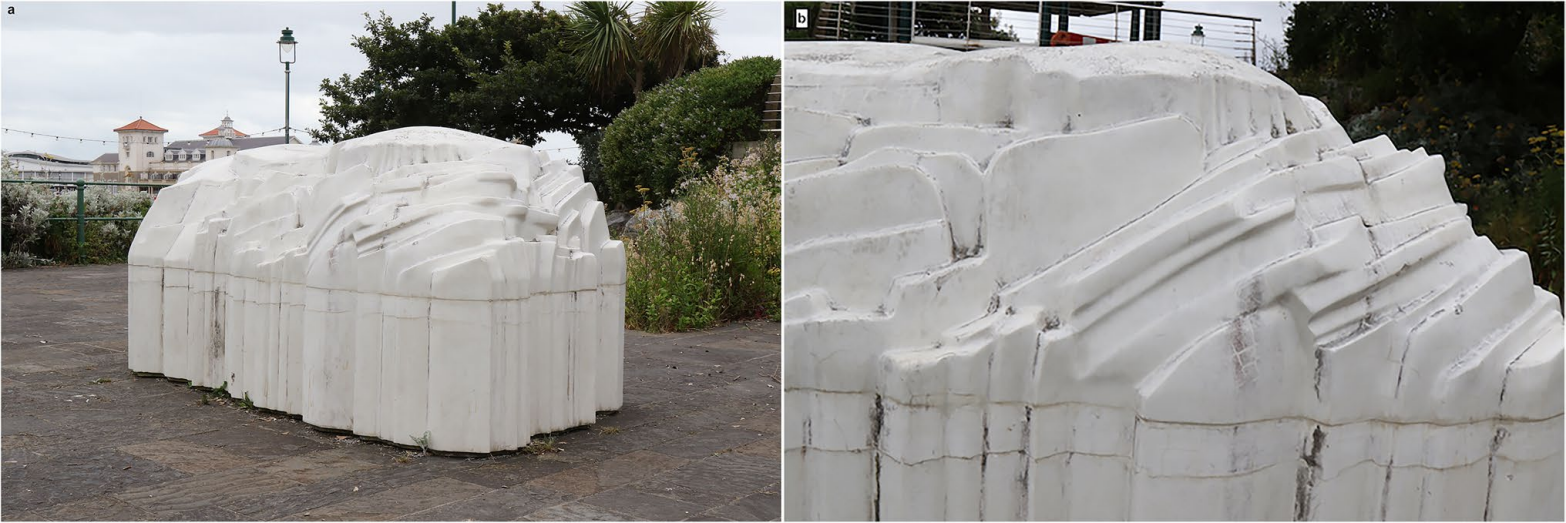
**a**, **b** General and detail views of Tania Kovats’ *Holm* at Madeira Cove [51.353528,-2.989751]. As with any sculpture, the viewer needs to be there and look around — and touch it. Its location within the local landscaping enables the viewer to approach the island. A small plaque (also in braille) gives a little information about the sculpture but is tucked away under branches. A more prominently placed small QR code would provide this and much more metadata. Photos: ©W.B. Whalley, 2022 CC BY-SA 4.0

Landscapes might be considered as visual representations or models in two or three dimensions. As well as a walk from Weston to Sand Point it is possible to walk to Steep Holm, or at least a sculpture representing it, by following the promenade to Madeira Cove at Weston. In the gardens is *Holm* (Fig. 7) by the artist Tania Kovats.^10^ Surprisingly, Holm does not look out over Madeira Cove to Steep Holm in the distance; the design brief was to utilise the area available. Kovats considers a bay within a bay, ‘almost fractal’, so that the stark white composition sits in the small area of the park. Figure 7b is a ‘stratigraphic’, closeup, view, but also provides a more geomorphological, landscape, view.

Aspects of looking carefully at any sculpture, sketch, painting or photograph are observing, thinking and reflecting (Whalley 2019). For the geomorphologist and geologist, Fig. 6a looks odd. It is not a detailed model, such as might have been produced by a lidar (laser scanned) digitization and 3-d printing, yet there is some broad geological structure and the skyline is similar to the view in Fig. 5. So, why odd? The ground level however is not, to me, ‘sea level’. Sea level is, or may be, represented by the horizontal line about halfway up the sculpture (Fig. 7a) and in the detail image (Fig. 7b). Below this ‘waterline’ is a series of ‘columnar structures’. The geology of the area suggests that this is unlikely to be the result of a sudden and unexpected (eustatic) draining of the sea. Rather, it is reminiscent of the uprights of certain types of oil/gas extraction platforms^11^ or the iron pillars supporting the Grand Pier (Fig. 5b). If the visitor examines Steep Holm at low tide by binoculars or by a visit, a notch can be seen just above sea level. This is due to the marine erosion and weathering in the last (approximately) 5000 years. Future sea level rise might bring little change to the view of Steep Holm but may require closing the tide gates on the esplanade (Fig. 4) more frequently: changes mean decisions.

Further questions for discussion at this location might include why didn’t the sculptor use natural rock, is it a scale model, does the rock of Steep Holm contain fossils, how are oil and gas extracted from rocks?

## Creativity: Circumscribing the Landscape View

To finish this brief tour of Weston and some of its wonders, we can return to forming our own landscapes. I suggested that both close and distant views are useful in placing objects in our perceived landscape. The landscape does not have to be the traditional ‘picturesque’ but posits various ways to observe and note the object under study, of which observational experience is perhaps the most obvious. The Claude glass (named after the French landscape painter Claude Lorrain) was much used by painters and indeed tourists in the 18th Century pursuing the picturesque-romantic aesthetic. Today, we have the mobile phone.

**Fig. 8:**
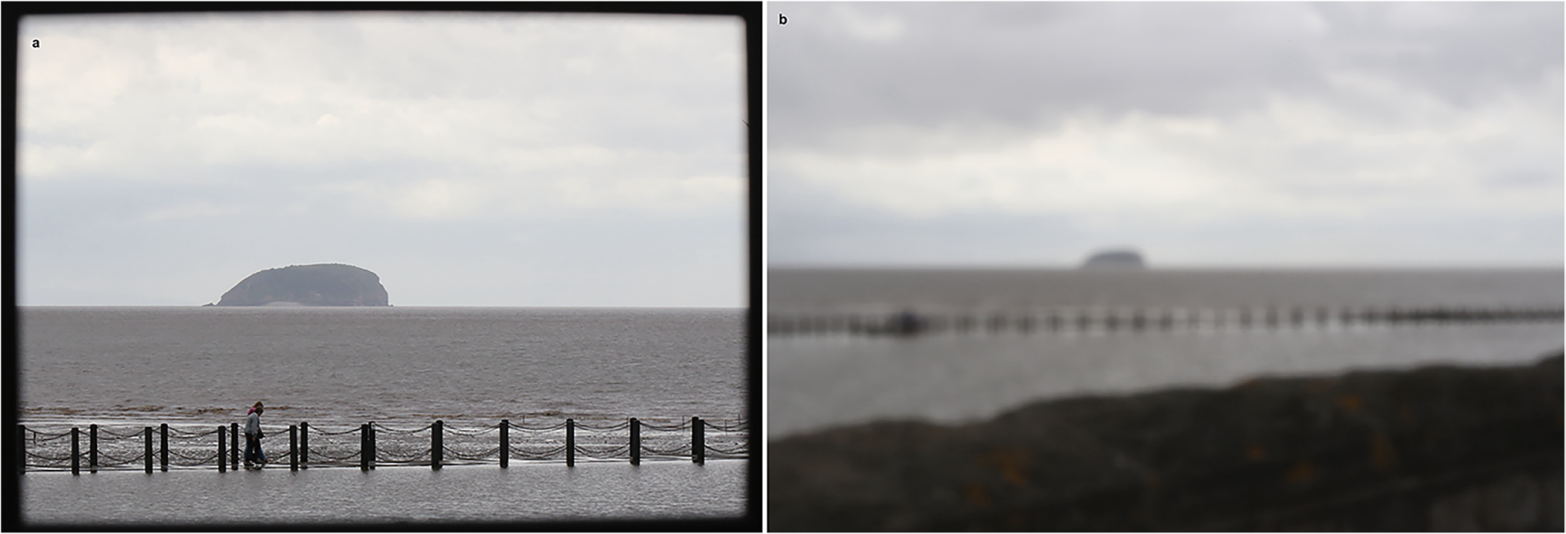
**a** Steep Holm from Madeira Cove at Weston-super-Mare, from [51.3533,-2.9898] and a direction of 260˚, i.e. summarized as {51.3533,-2.9898,260}. The walkers are on a causeway along which prams can be pushed and where there is wheelchair access. **b** Pinhole camera view of Steep Holm from Madeira Cove sea wall {51.3533,-2.9898,260}. Photos: ©W.B. Whalley, 2022 CC BY-ND 4.0

Figure 8a shows Steep Holm from Madeira Cove with a surrounding frame. The frame in this case was a black picture frame supported on a tripod. The image was focussed at infinity with the frame out of focus. The frame concentrates the image and forces the viewer to observe better and interpret the scene^12^. Figure 5b could be enhanced by post processing and cropping the pier on the left and even perhaps Steep Holm to concentrate on the textural differences of weathered stone wall and stainless-steel rail. There is no ‘right’ or ‘wrong’ view, rather the importance is on the cognitive processes involved in observing and explaining and merging aspects of art and science. From this point of view perhaps, landscapes *are* duplicitous.

## Visualising and Feeling Texture

The previous sections have concentrated on visual aspects of shore and off-shore features, whether natural or human artefacts. Observations focus thought and enquiry, and can be enabled by sketching, painting, photography or sculpting the features seen. The ‘walk’ described allows a ‘person on the prom’ to gain, not only points of view and locations of interest, but also something about the history, natural, social and geological, of the area. Our assumption, however, is of a fully sighted person. Visitors to the seafront may not necessarily be sighted. On the day before the sand sculptures (Fig. 3) are bulldozed into shoreline oblivion, local blind people are encouraged to visit and touch the sculptures. Nicola Wood, sculptor and co-organiser of the event, said ‘We believe an appreciation of art and an enjoyment for sculpture should not be limited to those who can see.’^13^ Sculptures, such as those in Fig. 3c, allowed visitors to feel the sand texture and this could be compared to the stones of the sea wall, thus posing the question, how does sand (Fig. 3) become a rock (Fig. 5)?

Figure 8b again depicts Steep Holm from Madeira Cove, this time attempting to show how (using a pinhole camera^14^ image) a person with visual impairment might see it. They might, however, be taken from that location, down, in a few steps to Kovats’ *Holm* and a tour could be taken in the cove-within-a-cove landscape conceived by the artist and landscape architects. It is possible to appreciate the sculpture by walking around it, pacing the distance from the ‘shore’ as well as touching the sculpture itself. Tactile examination of *Holm*, made of smooth-finished concrete, is not of course the same as the limestone of the original. The smooth finish is more akin to marble than limestone where weathering by rainwater, produces rough rock surfaces, soils and vegetation. The rough and jointed limestone has vegetation from plants to lichen; only the latter is on the sculpture itself. *Holm* is a visual representation and cognitive model rather than the output of a geomorphological process model. The interpreter needs to be aware of both the intentions of the artist as well as geological ‘truth’. Aesthetically, both are valid, but rather different, points of view. We then fall into a cognitive trap of using visual rather than visual plus haptic terminology in our observations. This an area of research still largely unexplored in the links between aesthetics and cognition (Currie et al. 2014), although Trevor-Roper (2012) informatively discusses art and defective sight.

However, we can re-run the tour for those with visual impairment, touching the honeycomb weathering and comparing that with the variety of weathering intensities. Visitors can feel the variation of coping stone weathering and its spatial changes. Use of the local limestone to produce quicklime for building can be seen at the kiln below the quarry at Uphill to the south of Weston Bay [51.32050,-2.98431].

Comparisons can also be made with smooth building stones, such as at and around the 8 m high, John Maine’s *Weston Arch*^15^ and the stone materials used for benches on the seaward side of the storm surge, ‘splash’ wall (Fig. 4). These benches are a mixture of Mendip limestone and polished/textured granites of various colours and provenances. Much of the promenade area in Weston provides observational opportunities for the visually impaired as well as the sighted. However, for these opportunities to be realised effectively, then appropriate metadata need to be supplied and be accessible to everyone.

## The Uses of Metadata

The term ‘metadata’, loosely ‘data about data’, may include the location of a photograph as well as the direction of view, date and time of day (Fig. 6). The geolocated data for some of the sites in this article provide locational information that can be related to the UK National Grid or *What3Words* systems. However, using decimal Latitude Longitude (dLL) provides easy location from a smartphone with a precision sufficient for everyday needs or via Google Earth (Whalley 2022) and for use in GIS. In the case of Kovats’ *Holm*, metadata could be the on-site information (included on the existing text and braille plaque) about the sculpture that is hard to find at the site. A web-search is required to find out more about the sculpture or of any of the geosites or concepts mentioned in this geo-promenade.

Changes in seafront features, such as buildings or weathering phenomena, over time as seen from picture postcards^16^ might best be shown by a comparison of metadata. But an image’s metadata also includes the artist or photographer as well as the title and other biographic information. This too may need to be searched and related to other ideas of interest. Conventional web searches are currently not good at making such connections, although new tools (the semantic web)^17^ are much better at using metadata. The eighteenth-nineteenth century polymath Alexander von Humboldt (1769–1859) in his ‘Kosmos’ suggested that:

'Those who observe from a reflective perspective recognize nature as a unity in diversity, as a connection of the manifold in form and mixture. The embodiment of natural forces and phenomena as a living whole.’ (Falk 2019)^18^.

**Fig. 9:**
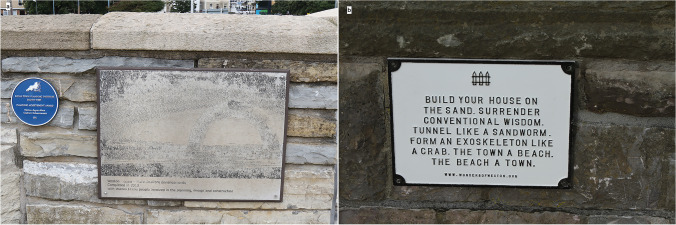
**a** Plaques on the sea wall at Weston. The blue plaque notes a Royal Town Planning Institute, RTPI, ‘South West Planning Achievement Award, Weston-Super-Mare Seafront Enhancement 2011’. The other is a rather sand-blasted (?) depiction of the seafront *Weston Arch* with a ‘Word Cloud’ of the arch comprising names as ‘thanks to the people involved in the planning, design and construction’. **b** One of a series of poems about Weston and the beach environment that are mounted on the sea wall. Photos: ©W. Brian Whalley, 2022 CC BY-SA 4.0

Natural history and earth science media programmes, no matter how expertly done and presented, lack the immediacy of active involvement and reflection that is suggested by Humboldt^19^. The promenade outlined here allows anyone to participate in exploration and in their own time. Forms of participation, whether stopping to look and think, share comments, sketch or take a photograph, are all valid, and indeed necessary, part of explaining; what, where, why and how-come? questions. Some of these may be answered on the spot by Humboldt’s ‘reflective perspective’ and, or even, by interrogating appropriate metadata. There are some locations on the promenade, such as in Fig. 9a, which say something about the site or, Fig. 9b, a short statement about the area. These are visible forms of object metadata, although searches on the web may provide further information and clues about interpretation. Using the geo-promenade helps direct visitors’ attention and not just pass them by.

Still more information could be gained by web-searches or in local newspaper and the availability of information (metadata) now available on smart devices is considerable. Most of the material covered in the footnotes is metadata appropriate to the geo-promenade. This, often local, knowledge does need to be curated for a geopromenade. Instead of a web link, the information could be provided by a QR code specifically as part of the exploratorium.

Most of the observations of objects or concepts, involving some combination of looking and thinking, made in this paper have associated metadata; about the object (such as Steep Holm and *Holm* and their settings), definite geomorphological concepts (salt weathering, flooding, mud) or perhaps vaguer ideas (romanticism, tourist, curiosity) as well as an artist’s website or even terms used (such as ‘vermiform rustication’). The need for metadata may depend on the purpose and take into account the age and background of participants and whether they are handicapped in some way. The metadata seen in Fig. 9a and b are limited but could be added to these locations (by a QR code for example) that would enhance the visit, or fieldtrip and its impact. Those unable to read or with visual impairment would benefit from alternative access to information via an embedded QR code where the text could be spoken. Thus, the visitor can bring together information about the objects and associated concepts in order to ‘explain’ them in some way. ‘Smart’, internet-linked, devices enable these exchanges to be achieved easily. Observations can clearly be linked by these metadata. This is what researchers (or police, lawyers, journalists and novelists) accomplish, as a matter of course, by training and practice.

Observations, ideas and conjectures are likely to be individual, even if taught in a simple ‘guide’, for example, as outlined in the first sections of this paper. These are likely to include, ‘how and why’ questions, in a loose sense, such as ‘how do tides work?’ and, ‘why did the council install the tide/splash gates’ are related but rather different. The first can be explained by reference to a textbook, although it is ‘because of the moon’ is not a satisfactory explanation. But mobile-equipped observers can take a quick dip into Wikipedia and use information there to understand better and progress beyond ‘lies to children’.

## The Behavioural Environment and Decision Making

**Fig. 10:**
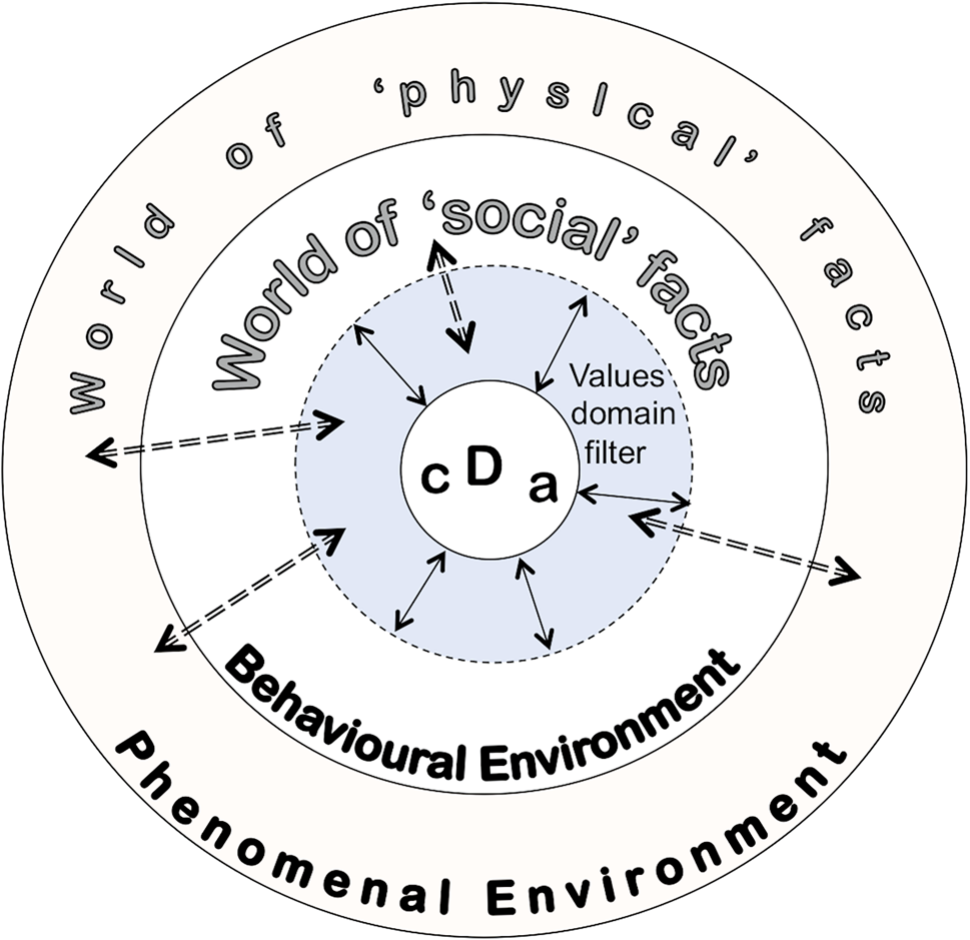
The phenomenal and behavioural environments of Kirk (1963) with modifications. The decision-maker D makes choices and decisions about facts and information in the ‘phenomenal environment’ by way of social and economic facts with filtering by values and operating in the ‘behavioural environment’. Decisions are rarely made alone but in conjunction with, for example, associates (a) on a project (such as the landscape architects for ‘Holm’ or collaborators (c) on a project or as a family group. The ‘values’ filter might be cost or aesthetics or even health and safety. Image: ©W. Brian Whalley, 2022 CC BY-SA 4.0

One tool to assist understanding in a complex world is via Kirk (1989) ‘behavioural’ and ‘phenomenal’ environments (Fig. 10). The topics in the first part of this paper might come under several academic topics; geography, geomorphology, geology, art, planning, engineering. Decisions need to be made, from the personal to the corporate: where to go today, where to take a photograph, how to examine weathering, how much money do we have to support a visit to Steep Holm, or commission a sculpture, how much will it cost to rejuvenate the coastal defences, what schemes should we use to produce electricity? These are not so much ‘research questions’ as ‘decision questions’. In the present context, there may be decisions related to aesthetics, cost and for environmental reasons. As such, they are worthwhile pointing out to the visitor. The proposal of a tidal barrier at Cardiff, across the Bristol Channel from Weston, is one such project which has long been under discussion^20^. Figure 10 is a schematic of Kirk’s concept which shows the breadth of decision making required within a world of ‘facts’ and knowledge. These are merely indicated in this discussion but are used to illustrate some of the environmental (in the widest sense) issues that are as relevant to geoheritage and geotourism as aesthetics.

A further aspect of behavioural and phenomenal environments that the exploratorium approach gets away from the formalities of learning and the traditional classroom approach to education. Furthermore, people like playing games and in particular, computer games. They also like quizzes (as opposed to examinations) and puzzles. These can be brought together, along with creativity by doing, or perhaps more generally, these things can be, fun. But probably not for everyone and certainly not all the time. People, especially young people, have different boredom thresholds in the behavioural environment. The concept of the outdoor exploratorium introduced here will need consideration from many points of view but with the capability for development by participants as much as organisers. Morris (2020) has recently argued for a better appreciation of sensory geographies which would be well explored further via exploratoria. Arts-based practices can be used as therapy, not least in music (Ledger and Edwards 2011), something not explored in this paper. Recent ideas by Kanngieser (2015) and the phenomenon of ‘sound’ make it relevant to decisions related to climate change as well as landscapes (Gallagher et al. 2016). Sound includes a poetic and linguistic view on climate change (Magrane 2020), so a geo-promenade is a good opportunity to extend and interrelate research interests for academics as well as the public good.

## Public Art: Objects and Earth Science Interpretations

**Fig. 11:**
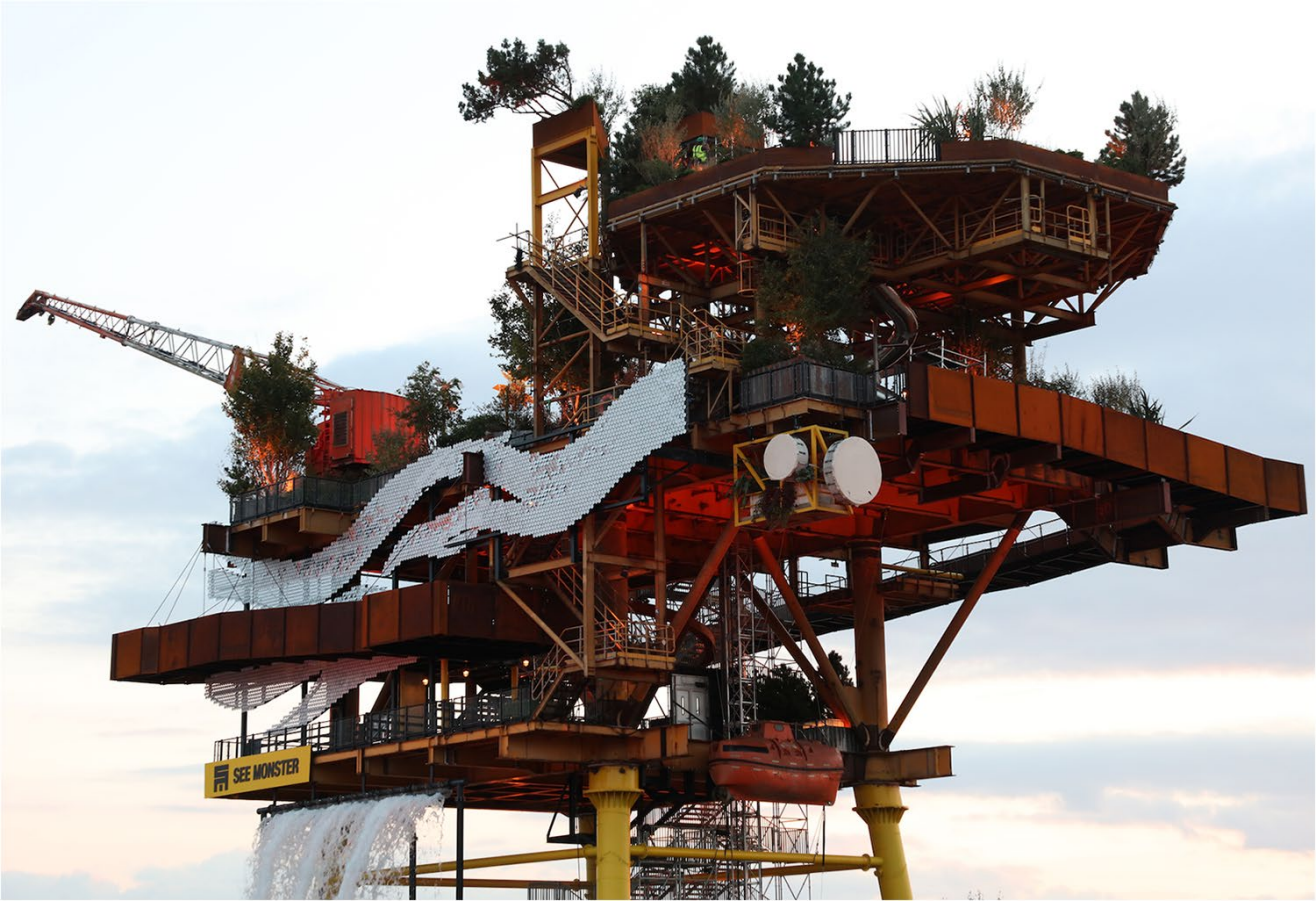
SEE MONSTER within the pool at the Tropicana, Weston-super-Mare. September 2022. The trees are additional to the original, decommissioned, off-shore platform! Water is being pumped to flow as a cascade (lower left). Photo: ©W. Brian Whalley 2022 CC BY-SA 4.0

Several stops on the geo-promenade are sculptures that involve geological materials; sand sculptures, Holm and The Weston Arch, as well as the natural weathering phenomena on walls. The sand is on the beach, Holm is concrete but the Arch is of ‘Chinese granite’, not of local provenance. Decision making presumably involved cost rather than identity, whether of place, local community or geology. Further discussion on the aesthetics of public art in the behavioural environment is necessary, not least to consider sustainability of transport. To the list of sculptures can be added (at least temporarily) SEE MONSTER (Fig. 11, Note 11) a decommissioned North Sea gas platform. This is another approach to geoheritage. Patrick O’Mahony, the artistic director of SEE MONSTER, considered that the project would serve as ‘a springboard to explore the concept of inherited structures and to question what we do with them’. Dr Ella Gilbert, British Antarctic Survey, climate science adviser to the project, said “See Monster is an opportunity to see and hear about the kind of solutions and possible futures we can create together and … to be excited by the science behind it and to learn how it helps us understand our planet.” (Note 11). This public art installation, as well being an entertainment that visitors can climb, can also provoke discussion about sustainability and the geological links between trees (as added to the structure) and renewable energy sources. As well as an installation, SEE MONSTER is an opportunity to present ideas on climate change, sea-level rise and energy sources, as described in this paper. It provokes ideas of a lighthouse, warning of hazards ahead. This is public art with an educational purpose and sits in well with the exploratorium concept. If it provokes discussion, all to the good.

## Discussion

Places are worth visiting from many points of view; objects can be seen or visualised but enjoyment can be extended by active involvement in a broadly educational experience. Observing, adding information, is necessary in geosciences and art and I suggest that they can be combined in ways that are part of the character of a location — a sense of place. As Arnheim (1980, 497) argued,

Everything we are learning about the mental functioning of scientists and artists strengthens the conviction that the intimate interaction between intuitive and intellectual functioning accounts for the best results in both fields. And the same is true for the average schoolchild and student.

But, as Lewis (1979, 26) indicates (as one of his ‘axioms for reading the landscape’), ‘The landscape does not speak to us very clearly. At a minimum, one must know what kinds of questions to ask’. The locations at Weston noted here are human artefacts as well as natural features but are geologically linked. The landscape underfoot or in front of a wheelchair is a geomorphological construct and the features are part of geo-heritage (Saunders 2012) and geo-information in the widest context (Gray 2013) as well as formal, subject-based, education. Although Lewis was concerned with the American cultural landscape, he noted that’Very few academic disciplines teach their students how to read landscapes, or encourage them to try. Traditional (sic) geomorphology and traditional plant ecology … were disciplines which insisted that their practitioners use their eyes and *think* about what they saw’ (Lewis 1979, 13). To look, observe and interpret need are part of answering the ‘understanding’ problem that compartmentalised educations invokes (such as passing examinations) rather than ‘appreciating’. Using open exploratoria is one way to counter specialisation. This involves cognition and psychology of aesthetics together with ‘science’ (Jacobsen 2006).

One major problem of understanding in earth sciences, as in astronomy, is time and especially ‘deep time’. Its significance can only be hinted at here, especially in the realm of decision making (Monroe 2012). However, ‘geo’ words are curriculum-based, along with art and landscape. The ‘out of classroom’, ‘out of textbook’ and’out of gallery’, but non-confrontational approach of this paper shows how objects and concepts can be linked via reflective perspectives and consilience. The information available or perhaps looked for (as in the phenomenal environment) can be associated with decision making (in the behavioural environment). By walking along and through a human-constructed and mediated geomorphological environment, the bricks, stone and concrete of the seafront can be explored by way of artistic creations, permanent or ephemeral. As Lancaster and Waldron (2020) argue, geologists can convey a sense of time and place in an artistic setting; this can be seen in the sand sculptures, weathering of stone wall caprocks as well as sculptures of by alluding to geological materials, locations and representations. These are as important as the impermanence of ‘Dismaland’ and the artistic-geomorphological sculptures of Richard Long (2015) and other land-artists, such as Kovats, in the presentation of public art. Like the honeycomb rock weathering at Weston (Fig. 5), Long’s ‘Sahara Circle’ is perhaps a long-term study in rock varnish formation (Whalley 2022).

This description of a geo-promenade locality does not include additional material such a geological timescale or images of small quartz grains or thin sections that might be useful for deeper exploration. But there is no reason these could not be supplied; for example, as paper handouts, downloadable pdfs or accessed on a website via a QR code or even as part of a local mobile app. It is here that the skills of museum and exhibition presenters should be engaged.

Tormey (2019, 197), in his consideration of education and knowledge transfer and provision of visitor information, considers that the choice of site depends on the nature and characteristics of the target audience and their flexibility. He lists criteria for the identification of sites for educational value:

i) Didactic potential: the capacity of a feature to be easily understood at different educational levels
ii) Geodiversity: number of different types of geodiversity elements present at the site
iii) Accessibility: access to the site in terms of difficulty and time spent for visitors
iv) Safety: related to the visiting conditions, consideration hazards and risks for visitors

and sites for geotourism/recreational based on the criteria:

i) Scenery: ‘visual beauty’ of the landscape or feature:
ii) Interpretative potential: the capacity of the feature to be easily understood and
iii) Accessibility: conditions of access to the site in terms of difficulty and time.

These criteria seem to be amply fulfilled at the geo-promenade described at Weston. However, decision making (Fig. 10) may involve more complex discussions than the geoheritage component alone by requiring attention to economic and social factors as well as aesthetic, considered for example by Pijet-Migoń and Migoń (2022).

## Conclusions and Future Research

Observation is not just ‘looking’, nor is it just aesthetics; craft, art, design and engineering all incorporate observations to elicit understanding 21. The promenade from Madeira Cove to the Grand Pier (Fig. 2) gives the visitor to Weston-super-Mare some exploratory ways of combining geoscience and art via observation. Sculpture is not ‘highbrow’ but can be related to everyday experiences of seeing and touching, such as the sea walls and promenade stone furniture and the ‘Weston Arch’. The engineering responses to sea-level rise and storm surges are incorporated within the aesthetics of a planned urban response to environmental changes. Education can, and should, be accompanied by understanding, even for everyday objects. However, ‘educating’ is a complex cognitive process, continuous for all ages. Exploratory learning, as demonstrated in this paper, provides new aspects, information, contexts and storylines about the commonplace. A visit to whatever might be perceived as ‘exotic’ — the non-quotidian experience (such as a seaside holiday), when supported with information and metadata, enables visitors to make their own documentary videos, sketches or photographs and thereby being involved in geoconservation (Gordon 2018). They can then place themselves in the role of town planner, artist, civil engineer as well as geoscientist. Any inquisitive tourist, including the academic, wanting to know more about a place can go on short journeys of discovery. These explorations can be done accessibly in an environmentally friendly manner. Looking out across the Bristol Channel and at the tide-surge protected town centre from the promenade brings home the ‘simplexity’ (Kluger 2008); simple effects but complex relationships to explain sea-level rise and global heating. Such perspectives might be explosive (Wainwright 2020, 211), ‘One essential task today is to create events and spaces that bring people together into a genuine confrontation with climate change—where they really face the crisis head-on’. Explanation, however, is more likely to be within a traditional geographical viewpoint (Hulme 2008) but importantly, should be within the domain of inclusivity and accessibility (Stokes et al. 2019). Exploratoria, such as the ‘geo-promenade’ described here provide important educational linkages between informal visits by tourists and local geology and geomorphology. In particular, they provide visual (and perhaps tactile) means of linking geoheritage landscapes to visual literacy (Whalley 2022). Using decimal latitude longitude (dLL) locations can be useful in locating places in the literature and also by adding to image metadata and enhancing the FAIR principles for data management: findable, accessible, interoperable, reusable (Wilkinson et al. 2016). Leeder and Lawlor (2017) use dLL to locate a variety of locations in their book, linking geology, landscapes, human occupation and art. Google Earth gives opportunities to link feet on the ground observations with diverse experiences. Mobile technologies are much more than media and social media devices, they can free users from traditional education boundaries and tap into learning for the future (Whalley et al. 2020, 2021). Gray (2019, Fig. 1) discusses ‘geosystem services’ that benefit society via geodiversity (topographies, geological materials and physical processes). This paper has shown that it is possible to incorporate discussion about such services as well as social policy making, economics and environmental awareness into ‘geo’ environments; geo-futures as well as traditional geology. Enhancing informed public debate about these pressures is vital for better decision-making about our geo-futures. Low-cost, pop-up, geo-promenades might be one way to help widen these debates as well as inspire geoscientists for the future (Gray 2019). Some of the ideas discussed here are being developed for broader geoscience training associated with fieldwork utilising mobile devices.

## Data Availability

The data used in this study are presented in the text. Images may be used under the CC licenses with acknowledgement.

## Footnotes

1 *As well as the recent book by *Brodie et al. (2019)*, Historic England has recently added material to its website by Johanna Roethe (*https://historicengland.org.uk/whats-new/research/back-issues/weston-super-mare-a-victorian-seaside-town/*) links to publications on Historical and Architectural Development and Heritage Action Zone reports. 'Weston-super-Mare: the view from above' by Edward Carpenter is also included in Historic England's Research Tourism, Seaside Special Issue, 2019, 42–49. Guidebooks generally say little about the geology of a place and that around Weston is particularly complex. The BGS iGeol app provides some basic map-based information. For those interested in geology *per se* to inspect the Lower Carboniferous fossils and volcanics at Middle Hope [*51.3919,-2.9573*] near Sand Point, north of Weston, see Faulkner, (1989). The early Carboniferous (Courceyan) Middle Hope volcanics of Weston-super-Mare: development and demise of an offshore volcanic high. Proceedings of the Geologists’ Association, 100, 1, 93–106.*

2 *'This is Silica; a bus shelter, kiosk, public artwork and electronic information display, all rolled into one': *https://thebeautyoftransport.com/2015/03/11/the-gleaming-spire-silica-weston-super-mare-somerset-uk/* and: *http://www.wolfgangbuttress.com/silica*. It is locally known as ‘the carrot’.*

3 *Wonders of Weston at: *http://www.situations.org.uk/projects/wonders-of-weston/*. Sea Change is an EU H2020 funded project that aims to establish a fundamental “Sea Change” in the way European citizens view their relationship with the sea, by empowering them, as Ocean Literate citizens, to take direct and sustainable action towards a healthy ocean, healthy communities and ultimately a healthy planet': *http://www.seachangeproject.eu/244-seachange/seachange-consortium-2

4 *Arthur Stanley Eddington, 'Science and the Unseen World'. A plaque on the wall at 42 Walliscote Road [51.34363,-2.97734] states, ' This house was the boyhood home of Arthur Stanley Eddington, one of the foremost scientists, not only of the twentieth century, but of the entire history of the human species. His achievements include, but are not limited to, the integration of modern astrophysics, major advances in the early history of relativistic cosmology, major additions to modern astronomical advancements, a superior exposition of the philosophy of physical science, and authorship of many books and articles making science more meaningful, not only to scientists, but to all rational and curious individuals. His achievements will radiate for the duration of the human species! Arthur Stanley Eddington 1882–1944'. The plaque, which is not the usual blue circular type seen in the UK, also contains some images of planets and the equation of 'Eddington Luminosity'. At one time, between the World Wars, he was as well-known as Albert Einstein and was a major follower of relativistic physics.*

5 https://en.wikipedia.org/wiki/Dismaland

6 https://en.wikipedia.org/wiki/Weston-super-Mare_Sand_Sculpture_Festival*. The sculpture area can be clearly seen in Google Earth imagery, 15 August 2016 at [51.3437,-2.9817]. A Wikimedia Commons entry is at: *https://commons.wikimedia.org/wiki/Category:Weston-super-Mare_Sand_Sculpture_Festival

7 *More information can be found at: *https://waterprojectsonline.com/wp-content/uploads/case_studies/2008/Weston-super-Mare-Sea-Defences-2008.pdf*. Unfortunately information from the contractors, Royal Haskoning DHV, has recently been removed. This shows the importance of curating knowledge sources for even a small exploratorium. Information about the sewage treatment works for Weston at [51.3054,-2.991] can be found at: *http://waterprojectsonline.com/case_studies/2008/EA%20Weston%20super%20Mare%20sea%20defence%202008.pdf*. Holiday makers probably rarely, if at all, think of the public works that need to be installed for a locality, let along the influx of visitors. The plant is part of Integrated Urban Drainage Management (IUDM) measures within the catchment. Such management schemes are related to sustainable drainage systems (SuDS). In the UK there are links to the underpinning earth science *via* the British Geological Survey; *https://www.bgs.ac.uk/geology-projects/suds/*.*

8 *New Scientist 2018, 3166, 24 February (*https://www.newscientist.com/article/2161693-mystery-honeycombs-in-rock-may-be-created-by-water-and-salt/*) includes an image. A recent A-Level textbook (*Bayliss et al. 2016*) mentions 'salt crystallisation' i.e. weathering (p. 71) but with no images and implies that it occurs only in 'hot deserts'. Wikipedia provides a much better description and explanation at *https://en.wikipedia.org/wiki/Weathering*. The geologist Archibald Geikie noted the weathering of gravestones in some Edinburgh churchyards in the 1880s and used dated headstones to gain a measure of stone weathering rates of the then highly polluted atmosphere, of 'Auld Reekie' (*Morgan 2016*). Grave headstones have been used to provide dates for lichenometry and simple identification could be added to the geosites described here. The Field Studies Council has produced a ‘Churchyard lichens guide’ (*https://www.field-studies-council.org/shop/publications/churchyard-lichens-identification-chart/*) one of several plastic-covered guides for field use.*

9 *Steep Holm has an informative Wikipedia site (*https://en.wikipedia.org/wiki/Steep_Holm*). The island is owned by the Kenneth Allsop Memorial Trust to commemorate the journalist and conservationist Kenneth Allsop (1920—1973; *https://en.wikipedia.org/wiki/Kenneth_Allsop*). Steep Holm was bought by public subscription in 1976 and is run as a nature reserve. Information is available on the Trust Websit**, *http://www.steepholm.org.uk/*.*

10 *Tania Kovats noted, ‘**Steep Holm**’s distinctive punctuation of the horizon … as a poetic eye catcher, mysterious and utopian, existing remotely as a protected wilderness’. The design brief and the placing of the sculpture as landscape narrative. Kovats' proposals for the gardens' renewal took inspiration from the distinctive characteristics and native plants of Steep Holm. Landscape architects Grant Associates developed these proposals and designed a new viewing platform for horizon-gazing at Madeira Cove with the sculpture HOLM at its centre. Native planting was re-introduced and Steep Holm's distinctive combination of layered rock formations and man-made concrete interventions have inspired the contemporary treatment of the site'. Information from: *http://www.fieldartprojects.com/index.php/artist/kovats*.*

11 *Typically, a multiple concrete deep-water structure (‘condeep’). In July 2022 SEE MONSTER, ‘the world's first and only repurposed North Sea* *offshore platform’, will be a tourist attraction on Weston seafront. ‘It will use its unique seafront location to harness the power of the elements to provide renewable energy for the structure, transforming the seafront into a large-scale public art installation. … With places to meet, be a fun and educational place which will focus on weather, climate change and green technologies … have a dedicated outreach team with a science, technology, engineering, arts and maths (STEAM) focus.’* (https://www.visitsomerset.co.uk/discover-somerset/towns-villages/north-somerset/visiting-weston-super-mare/festivals-and-events-in-weston-super-mare/see-monster-arrives-at-weston-super-mare-in-2022). Further information on this ‘Unboxed, creativity in the UK’ project is at https://www.bbc.co.uk/news/entertainment-arts-58980615 and at https://www.bbc.co.uk/news/uk-england-somerset-63004739.

12 *Permanent small picture frames have been used to view parts of the landscape, for example at Spurn in E Yorkshire [53.6110,0.0145] and at Alnwick Castle, Northumberland [5.4160,-1.7051]. The latter helps to illustrate the change in English landscapes produced by Lancelot 'Capability' Brown by comparison with old maps. Smartphone apps can produce such framing effects but these are post processing rather than the frame assisting observation in the field.*

13 *The original link (*http://www.thewestonmercury.co.uk/news/weston-sand-sculpture-festival-extends-its-stay-1-521960*)* now missing, shows the need for curation of interpretative metadata,

14 *This was done by replacing the lens on a DSLR, with a pinhole and recording the image digitally but apps available for smartphones simulate this effect.*

15 *At [51.35161,-2.98455] not viewed directly above but the engineering associated with it can be seen at another metadata point, *http://www.trscaffolding.com/case-studies/weston-super-mare-stone-arch*. It was designed by John Maine RA and built of 'Chinese granite'; *http://www.waymarking.com/waymarks/WMDKK1_The_Weston_Arch_Knightstone_Road_Weston_Super_Mare_Somerset*. It was integrated into the seafront defences as part of the 'Sea Change' initiative in the town. See also Note 1.*

16 *For an example of images linked to metadata supplied by a poem on YouTube, see *https://www.youtube.com/watch?v=uivveQwoslc* and see also Note 1 and *Butt (2018)*.*

17 *The semantic web (sometimes known as Web 3.0) and its use is outlined in a paper by *Berners-Lee et al. (2001)*.*

18 *'Die Natur ist für die denkende Betrachtung Einheit in der Vielheit**, **Verbindung des Mannigfaltigen in Form und Mischung**, **Inbegriff der Naturdinge und Naturkräfte**, **als ein lebendiges Ganze.' Humboldt, A. v. (1845)**: **Kosmos**, **Entwurf einer physischen Weltbeschreibung. Vol. S.1, p. 6. Translation from Falk, G.C. (2018)*.

19 *Although not obvious geosites, litter (trash) bins on the promenade and seafront remind the visitor of the significance of plastic pollution and its global oceanic spread. At a local level, this needs to tie into the need for education and litter collectors, sustainability, geoexploration and the petrochemical industries.*

20 *For basic information on the history of this project see: *https://en.wikipedia.org/wiki/Severn_Barrage*. The much smaller Swansea Bay Barrage scheme came under discussion in the context of a mixed power economy which relates to the proposed Hinkley Point C nuclear power station 20 km south west of Weston. The project’s website (*www.tidallagoonpower.com/projects/swansea-bay/*) also has a list of comparative new build costs for power stations. Planning consent expired in spring 2020 after the UK government failed to support the project. The energy crisis of spring 2022 has now brought some of these issues back into focus with respect to subsidies for nuclear power being added to domestic electricity bills. It is also worth noting in the context of tidal range and onshore storms that a seawall and ‘wave returner’ is being built and also that dredging of mud in the Bristol Channel is necessary, specifically for Hinkley Point C.*

21 ‘Planes fly high overhead, cars stream by. Phone screen flash, shop signs call out to us and adverts offer their wares. Images jostle with each other, objects line up for inspection, while time marches on regardless. We don’t stop to look at much anything nowadays.’ Ward (2014, p. 7) Ways of Looking.
